# Freshwater microalgae *Nannochloropsis limnetica* for the production of β-galactosidase from whey powder

**DOI:** 10.1038/s41598-024-65146-6

**Published:** 2024-06-21

**Authors:** Yuchen Li, Svitlana Miros, Hans-Georg Eckhardt, Alfonso Blanco, Shane Mulcahy, Brijesh Kumar Tiwari, Ronald Halim

**Affiliations:** 1 School of Biosystems and Food Engineering, University College Dublin, Belfield, Dublin 4, Ireland; 2 UCD Conway Institute, University College Dublin, Belfield, Dublin 4, Ireland; 3 School of Chemistry, University College Dublin, Belfield, Dublin 4, Ireland; 4 Arrabawn Co-Operative Society Ltd., Nenagh, Co. Tipperary Ireland; 5 Department of Food Chemistry and Technology, Ashtown Teagasc Food Research Centre, Dublin 15, Ireland

**Keywords:** Dairy processing, Microalgae, *Nannochloropsis*, Whey powder, Bioremediation, Sustainable wastewater treatment, Algae–bacteria interaction, Bioremediation, Chemical engineering

## Abstract

This study investigated the first-ever reported use of freshwater *Nannochloropsis* for the bioremediation of dairy processing side streams and co-generation of valuable products, such as β-galactosidase enzyme. In this study, *N. limnetica* was found to grow rapidly on both autoclaved and non-autoclaved whey-powder media (referred to dairy processing by-product or DPBP) without the need of salinity adjustment or nutrient additions, achieving a biomass concentration of 1.05–1.36 g L^−1^ after 8 days. The species secreted extracellular β-galactosidase (up to 40.84 ± 0.23 U L^−1^) in order to hydrolyse lactose in DPBP media into monosaccharides prior to absorption into biomass, demonstrating a mixotrophic pathway for lactose assimilation. The species was highly effective as a bioremediation agent, being able to remove > 80% of total nitrogen and phosphate in the DPBP medium within two days across all cultures. Population analysis using flow cytometry and multi-channel/multi-staining methods revealed that the culture grown on non-autoclaved medium contained a high initial bacterial load, comprising both contaminating bacteria in the medium and phycosphere bacteria associated with the microalgae. In both autoclaved and non-autoclaved DPBP media, *Nannochloropsis* cells were able to establish a stable microalgae–bacteria interaction, suppressing bacterial takeover and emerging as dominant population (53–80% of total cells) in the cultures. The extent of microalgal dominance, however, was less prominent in the non-autoclaved media. High initial bacterial loads in these cultures had mixed effects on microalgal performance, promoting β-galactosidase synthesis on the one hand while competing for nutrients and retarding microalgal growth on the other. These results alluded to the need of effective pre-treatment step to manage bacterial population in microalgal cultures on DPBP. Overall, *N. limnetica* cultures displayed competitive β-galactosidase productivity and propensity for efficient nutrient removal on DPBP medium, demonstrating their promising nature for use in the valorisation of dairy side streams.

## Introduction

Dairy products, such as milk, cheese and yogurt, are staple agricultural products worldwide. The European Union is one of the largest producers and markets of dairy products in the world, playing a dominant role in the manufacturing and consumption, of cheese products^1^. However, dairy processing and cheese production generate considerable amounts of nutrient-rich wastewater and by-products (such as whey) which are often subjected to further treatment using ultrafiltration to produce protein concentrate and permeate^1–3^. The resulting whey permeate is rich in dissolved nutrients, such as lactose, soluble proteins, and lipids and often has a high phosphate content^4–7^. Dairy side streams pose serious environmental problems due to high organic load, resulting in a high level of Chemical Oxygen Demand (COD) and Biological Oxygen Demand (BOD)^1,8^. Conventional physicochemical technologies to treat dairy side streams prior to disposal to water bodies include a series of mechanical and chemical steps, such as electrochemical^9^ and coagulation–flocculation^10^ methods. Although these methods are effective in removing emulsified compounds^6^, the high demands for additional chemical and energy and their low elimination rate of COD add significant environmental burden to dairy manufacturing. The linear ‘treat-and-discharge’ business model of conventional waste treatment processes also do not generate any useful products that can contribute to profit margin, accruing a net economic cost to dairy manufacturers and depriving any commercial incentives for continuous process improvement. Therefore, developing a novel technology able to successfully upcycle the nutrients in dairy side streams into higher value products is of critical importance to enhance both the environmental sustainability and commercial return of dairy product manufacturing. For the EU, a circular dairy system can contribute to industry expansion resulting from EU-wide 2015 abolition in milk quota production.

Microalgae are considered as a promising feedstock utilised in several fields, such as sustainable biofuel production^11–13^, nutrition for human and animal^14,15^, and aquaculture^16^, due to their high areal productivity^17^. In addition, microalgae are a source of high-value bioactive compounds (e.g., lipids, proteins, pigments and enzymes)^15,17^, which has significant industrial applications, especially in food and nutraceutical sectors^18,19^. The application of microalgae biotechnology for the treatment of dairy side streams^11,20^ has recently garnered interest for its potential to achieve simultaneous bioremediation and co-generation of useful biomass/products that can be recycled in a circular economy model. Microalgae represent an environmentally sustainable and commercially attractive process to handle dairy side streams, having the potential to transform excess nutrients into higher-value biochemicals with minimal chemical and energy requirements, while contributing to carbon circularisation through photosynthetic capture of point CO_2_ emission. The majority of studies investigating the application of microalgae biotechnology in treating dairy side streams have focused their work on a few model organisms, such as *Chlorella* sp. and *Scenedesmus* sp.^12,21,22^ , due to their propensity for high/rapid biomass productivity. The use of *Nannochloropsis* for the valorisation of dairy side streams has to-date only been explored in a couple of studies, both of which are derived from our prior investigation^3,4^.

Species belonging to the *Nannochloropsis* genus are generally rich in eicosapentaenoic acid (EPA) ^23,24^, a type of omega-3 polyunsaturated fatty acids (ꞷ-3 PUFAs) that is ubiquitously integrated in food and dairy products, animal feed and aquaculture feed for nutrient enrichment as high-value ingredient to promote immunity and brain development. Fish oil derived from wild-caught fish is the current primary source of ꞷ-3 PUFAs. *Nannochloropsis* biomass can therefore be used as novel feedstock for ꞷ-3 PUFA production in replacement of fish oil, alleviating pressure on the marine ecosystem^25^. The cost of producing *Nannochloropsis* biomass can be lowered through the use of agri-food side-streams as nutrient-rich culture medium (such as whey permeate) instead of standard medium synthesised from commercial fertilisers^26^. Our previous study has shown that *Nannochloropsis* is able to assimilate lactose directly by secreting extracellular β-galactosidase^3,15^, eliminating the need for treating dairy side streams with chemicals/enzymes to stimulate lactose hydrolysis prior to microalgae cultivation and thus simplifying the design of microalgae system for the treatment of dairy side streams. β-galactosidase is a valuable precursor in the formation of prebiotic compounds, such as galactooligosaccharides (GOS) in infant formulas^18^. The application of *Nannochloropsis* for the treatment of dairy side streams can thus provide multilayered commercial benefits to dairy manufacturers through the displacement of conventional wastewater treatment technologies and the co-generation of EPA and β-galactosidase enzyme.

However, our current understanding of the mechanism responsible for lactose assimilation in *Nannochloropsis* is based on cultivation using lactose-enriched standard growth medium^3,15^; *Nannochloropsis* capacity for lactose metabolization on actual dairy side streams has never been investigated. It is possible for the dairy processing side streams to contain toxic contaminants that have inhibitory effects on the growth of microalgae^15^.

In addition, the need to cultivate microalgae under axenic conditions in order to prevent bacterial takeover is a critical bottleneck for commercial application of any microalgae-based wastewater treatment (including those envisaged for the treatment of dairy side streams). Even though thermal sterilisation methods (e.g., autoclaving) can be used as a mechanical pre-treatment step to maintain culture axenicity in lab-scale experiments, the high energy consumption and capital costs associated with these methods preclude their application at industrial scale. A possible solution is to deploy sterilisation methods with lower energy and infrastructure cost (such as cross-flow microfiltration) and/or to understand specific microalgae–bacteria interaction in given dairy side streams and harness this synergy to enable stable consortium. Previous studies investigating the cultivation of microalgae–bacteria symbiotic consortium in dairy wastewater^27,28^ have not investigated microalgal-bacteria interaction in *Nannochloropsis* cultures. There are two types of bacteria: contaminating bacteria from the dairy side streams and associated/native bacteria that are present as part of microalgal phycosphere transferred into the culture from non-axenic culture inoculum.

Finally, since most of the known *Nannochloropsis* species are marine species, their growth in dairy side streams can be hampered by low salinity of the medium^29–31^. To bypass the need for salinity intervention, this study has investigated the application of freshwater *Nannochloropsis* species *N. limnetica*. To the best of authors’ knowledge, this is the first-ever study to report on the cultivation of the freshwater *N. limnetica* on dairy side streams or any wastewater.

This study investigated the potential of microalgae belonging to the *Nannochloropsis* genues (i.e., *N. oceanica* and *N. limnetica*) for the valorisation of lactose-rich dairy processing by-product (DPBP) into valuable biomass and β-galactosidase enzyme. *N. oceanica* was not able to grow on the DPBP medium, allowing the investigation to concentrate solely on *N. limnetica.* Experiments in the study were devised to derive the following insights on the potential of *N. limnetica* for the treatment of dairy side streams: (1) the capacity of *N. limnetica* to grow on DPBP medium, (2) the need of pre-treatment regimes on microalgae–bacterial population dynamics, (3) the production of β-galactosidase enzyme and the extent of nutrient bioremediation in the DPBP medium, and (4) the role that microalgae–bacteria interaction plays in driving overall system performance. *N. limnetica* was cultivated on both autoclaved and non-autoclaved DPBP media and their biomass production was compared. The levels of microalgal population and bacterial population across all cultures were quantitatively measured using flow cytometry combined with microscopic and staining methods and correlated with system performance to dissect the role of algae–bacteria interaction. The activity of extracellular β-galactosidase and sugar concentration (i.e., lactose, glucose and galactose) in the cultures were monitored to determine the mechanism of lactose assimilation. Finally, the changes of different nutrients (nitrate, phosphate and protein) in the different cultures were tracked to determine bioremediation efficiency.

## Materials and methods

### Microalgae strains and dairy waste

*N. oceanica* (CCAP849/10, isolated from operational hatchery western Norway) was obtained from the Culture Collection of Algae and Protozoa (CCAP, Scottish Association for Marine Science, Oban, Scotland, U.K.). *N. limnetica* (SAG 18.99, isolated from village pond, Sachsen-Anhalt, Schwarz, Germany) was obtained from the Culture Collection of Algae at Göttingen University (SAG, Germany). Dairy processing by-product (DPBP) or whey powder was collected from Arrabawn Co-Operative Society Ltd., Co. Tipperary, Ireland. Liquid whey was subjected to series of clarification, evaporation, and spray drying steps to produce whey powder. Prior to the experiment, whey powder was added to Milli-Q water at 5% (w/v) and dissolved via agitation to produce DPBP medium. The solution was centrifuged at 10,000 rpm for 15 min to settle any insoluble particles and then divided into two separate aliquots; one was autoclaved at 121 °C for 15 min while the other was not subjected to any form of sterilisation. Both the autoclaved and non-autoclaved solutions were collected and employed as DPBP growth media in this study. The physicochemical parameters of the autoclaved and non-autoclaved DPBP media are presented in Table 1.

**Table 1:** Characteristics of autoclaved and non-autoclaved DPBP medium. *n/a = not available.

	Nitrate (ppm)	Nitrite (ppm)	Ammonium (ppm)	Soluble proteins (ppm)	Phosphate (ppm)	Total soluble nitrogen (ppm)	N/P ratio	Lactose (g L^−1^)	Salinity (parts per thousand)
Non autoclaved DPBP medium	14.86 ± 0.67^a^	1.45 ± 0.03^a^	4.56 ± 0.65^a^	73.89 ± 2.18^a^	200.77 ± 0.12^a^	19.17 ± 0.71^a^	0.29	45.52 ± 0.94^a^	2.67 ± 0.03^a^
Autoclaved DPBP medium	22.90 ± 0.90^b^	1.42 ± 0.07^a^	29.62 ± 3.92^b^	53.65 ± 2.93^b^	126.48 ± 0.58^b^	37.15 ± 2.55^b^	0.90	45.98 ± 0.19^a^	2.47 ± 0.11^a^
f/2 medium	54.71	n/a*	n/a*	n/a*	3.44	12.36	11.02	n/a*	35
Bold Basal’s medium (BBM)	182.38	n/a*	n/a*	n/a*	153.34	41.21	0.82	n/a*	0.4

All results are reported as mean of n ± std. n = 4. Statistical tests were applied on differences in nitrate, nitrite, ammonium, soluble proteins, phosphate, total soluble nitrogen, lactose and salinity between non-autoclaved medium and autoclaved medium. If the values for a particular parameter are significantly different between the two media (p < 0.05), they are denoted with different letters in the column (a or b). If the values for a particular parameter are not significantly different between the two media (p > 0.05), they are denoted with the same letters in the column (a).

### Culture conditions and growth parameters

Microalgal inoculum cultures were cultivated in 300 mL of f/2 medium^32^ for *N. oceanica* or Bold’s Basal medium^33^ for *N. limnetica* at ambient temperature (22 °C ± 1 °C) for 14 days. All growth experiments were performed in a 2 L bioreactor with a 1.5 L working volume. Microalgal inoculum was transferred to autoclaved (A) or non-autoclaved (NA) DPBP solution obtained from the previous section to produce culture with a starting OD_750_ of ca. 0.1. Blank DPBP media without microalgae inoculation were monitored as control groups, leading to a total of 5 independent experiments: A (autoclaved DPBP medium without microalgal inoculation), NA (non-autoclaved DPBP medium without microalgal inoculation), A-NO (*Nannochloropsis oceanica* cultivation on autoclaved DPBP medium), A-NL (*Nannochloropsis limnetica* cultivation on autoclaved DPBP medium), and NA-NL (*Nannochloropsis limnetica* cultivation on non-autoclaved DPBP medium). Cultures were cultivated under constant stirring (200 rpm) on a 16 h:8 h light:dark cycle with light intensity of 220 μmol m^−2^ s^−1^ and constant air sparging of 5 L min^−1^ for 8 days. Temperature of the cultures was maintained at 22 °C ± 1 °C. Air compressor was used to deliver air into each bioreactor. An inline 0.2 μm filter was employed in the air supply line of each bioreactor to eliminate potential bacterial contamination originating from the compressed air. Evaporation level throughout the cultivation period was monitored by weighing of the growth system at regular intervals. A total evaporation loss of 4.20% ± 0.38% of the working volume was recorded across all cultures at the end of the 8-day cultivation period. All cultivations were performed in duplicate.

Biomass concentration of the culture was measured by vacuum filtration using 0.45 µm pore size cellulose filter. 10 mL sample was collected from each culture on daily basis. The sample was filtered and rinsed several times using distilled water; the filter kept in the oven at 80 °C for two days until constant weight. Maximum specific growth rate (µ_m_, day^−1^) and maximum biomass concentration (Y_m_, g L^−1^) were calculated using Algal Data Analyser (ADA) software^34^ as outlined in our previous study^3^.

### Flow cytometry

Cultures were sampled every two days for flow cytometry analysis. 1 mL of culture was centrifuged at 10,000 rpm for 10 min and the supernatant was carefully removed. Then 500 µL Dulbecco's phosphate-buffered saline (PBS) and 500 µL 4% paraformaldehyde were added to the pellet and vortexed to fix the cells. SYBR™ Green I (SG) was employed as a nucleic acid stain for both microalgae cells and bacteria cells in this study. The dye was first diluted 1000 times in anhydrous dimethyl sulfoxide (DMSO). Our preliminary method development has shown that high level of dilution was needed to achieve good staining efficacy for nucleus fluorescence determination. 28 µL of dye solution was then added to 250 µL of fixed algal suspension, vortexed for 30 s, and incubated at ambient temperature for 3 min to make sure the stain went into the cells. The fluorescence intensity of the suspension was measured using CytoFLEX LX flow cytometer. SG fluorescence was excited using blue laser and collected by emission at 525 nm^35^. Signals were analysed for both forward scatter and side scatter. Flow cytometry analyses of every sample was completed on the same day of sample collection to ensure that accurate representation of microalgal and bacterial populations were captured. The data were then processed using CytoFLEX software under logarithmic scales to determine cell population dynamics.

### Bacterial isolation and microscopy

To observe the morphology of the bacterial population, bacterial cells from the cultures were isolated and subjected to gram staining. For bacterial isolation, 1 mL of microalgae culture in DPBP medium on day 8 of cultivation (A-NL and NA-NL) was serially diluted by tenfold; 100 μL aliquots of each dilution were spread onto Marine Broth (MB) agar plates and incubated at 25 °C for 5 days. Bacterial colonies with different morphology were then carefully isolated and streaked on MB agar plates. Microscopy slides were prepared from the isolated bacterial colonies. Gram staining was performed with a Gram staining kit, using crystal violet as the primary stain, iodine solution as the mordant, acetone/ethanol (50:50 v:v) as the decolourizer, and 0.1% safranin solution as the counterstain^36^. The slides were examined under a microscope at a 60 × objective magnification in oil immersion.

### Chemical analysis of DPBP medium

15 mL of each culture was sampled every day for analyses of nitrate, nitrite, ammonium, phosphate and protein concentration, sugar analysis (using high performance liquid chromatography HPLC), and β‑Galactosidase assay. The sample was centrifuged at 4000 rpm for 15 min; the resulting supernatant carefully isolated using a syringe and filtered through 0.22 µm nylon membrane to remove any biomass. The supernatant was then stored at − 20 °C while awaiting for analysis.

Commercial kits were employed to quantify the concentration of nitrate, nitrite, ammonium, phosphate, and protein in the supernatants. The nitrate, nitrite and ammonium kits were obtained from API (Mars Fishcare, USA), phosphate kit obtained from Hach, and Bradford reagent for protein determination obtained from Merck. Chemical analysis for each collected sample was carried out in duplicate. All chemical analyses were carried out according to suppliers’ instructions.

Total soluble nitrogen (TSN) in the medium was calculated using Eq. (1).

1 $${\text{TSN}}\left( {{\text{ppm}}} \right) = C_{{{\text{NO}}_{3} }} \times \eta_{1} + C_{{{\text{NO}}_{2} }} \times \eta_{2} + C_{{{\text{NH}}_{4} }} \times \eta_{3} + C_{{{\text{protein}}}} \times \eta_{4}$$

where $C_{{{\text{NO}}_{3} }}$, $C_{{{\text{NO}}_{2} }}$, $C_{{{\text{NH}}_{4} }}$ and $C_{protein}$ were the nitrate, nitrite, ammonium and protein concentration (ppm) respectively, and $\eta_{1}$ ($\eta_{1} = 0.23$), $\eta_{2}$ ($\eta_{2} = 0.30$), $\eta_{3}$ ($\eta_{3} = 0.78$) and $\eta_{4}$ ($\eta_{4} = 0.16$) are the nitrogen content of each component.

### HPLC analysis and β‑galactosidase activity assay

Lactose, glucose, and galactose concentrations in the supernatant were determined by HPLC (Agilent Technologies 1200 Series) equipped with a refractive index detector (G1362A RID) and an Agilent ZORBAX ­NH_2_ (250 mm × 4.6 mm containing 5 µm silica particles) column. A mixture of acetonitrile and HPLC grade water was used as eluent at an isocratic flow rate of 2 mL min^−1^ and constant temperature of 35 °C. The injection volume was 10 µL with a column temperature at 30 °C and a running time of 9 min. Sugars were identified by retention time comparison with standards and quantified by peak area against a linear calibration curve of the standards.

The β-galactosidase activity in the supernatant was measured using *o*-nitrophenyl-*β*-d-galactopyranoside (ONPG) assay^37^ based on the method outlined in our previous study^3^.

### Statistical analysis

A total of four measurements were carried out for all chemical analyses (2 biological replicates × 2 analytical replicates). Data in this study were expressed as mean ± standard deviation (SD). Statistical analysis was performed using SPSS statistical (version 27.0.1, IBM Inc., Chicago, IL, USA). One-way analysis of variance (ANOVA) was performed on each parameter using Post Hoc with Tukey’s HSD test with variables reported as significant at 95% confidence (p-value < 0.05).

## Results

### Nutrient composition of DPBP medium and effect of autoclaving

As shown in Table 1, the total soluble nitrogen concentration of the non-autoclaved 5% (w/v) whey powder solution (or DPBP medium) were within range of standard media formulations for both marine (f/2) and freshwater (BBM) microalgae, signifying the side stream’s potential to support microalgal growth. However, since the salinity of the DPBP medium was considerably lower than that of seawater or f/2 medium, successful growth of any marine microalgal species on the medium will likely require prior salinity adjustment.

Sterilisation of the DPBP medium via autoclaving led to phosphate precipitation which reduced the soluble phosphate content of the medium by ca. 37% (from 200.77 ± 0.12 to 126.48 ± 0.58 ppm) and a simultaneous release of nitrate and ammonia (likely ascribed to protein breakdown) which increased the total soluble nitrogen of the medium by ca. 94% (from 19.17 ± 0.71 to 37.15 ± 2.55 ppm). The compositional differences between the autoclaved medium and non-autoclave medium were found to be statistically significant (p < 0.05). Overall, autoclaving resulted in a threefold increase in the N:P ratio of the medium from 0.29 to 0.90.

### Microalgae growth on DPBP

**Figure 1:**
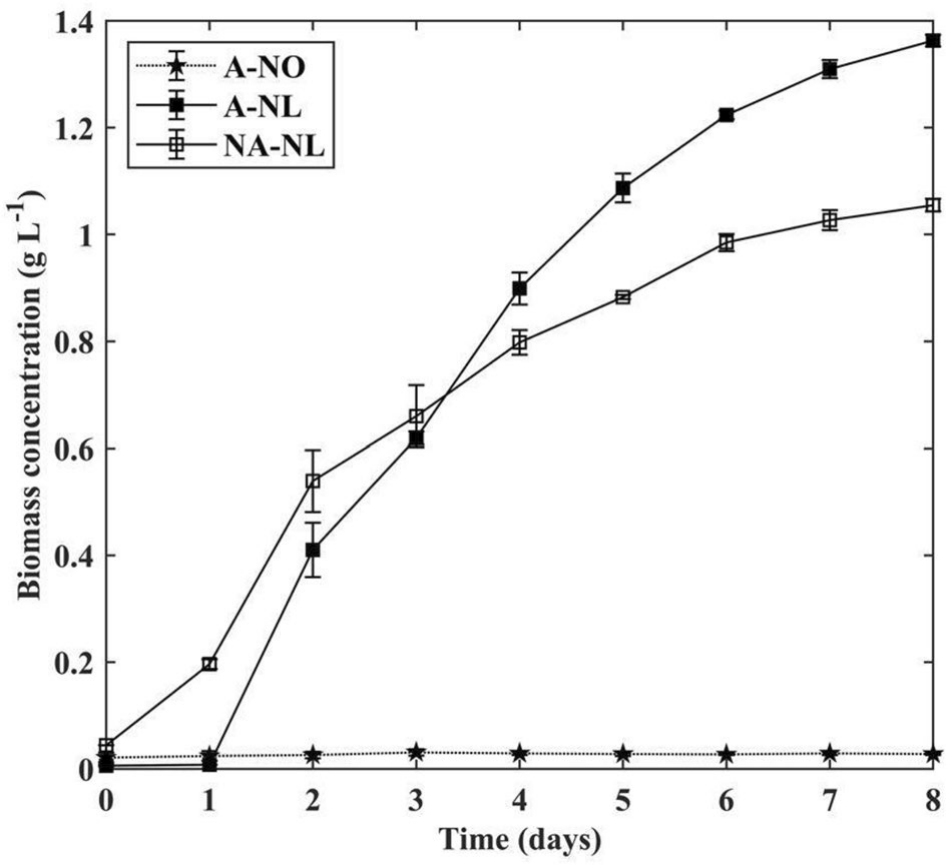
Biomass concentration of *Nannochloropsis* cultures on autoclaved and non-autoclaved DPBP medium. A-NO: *N. oceanica* cultivation on autoclaved DPBP medium, A-NL: *N. limnetica* cultivation on autoclaved DPBP medium, and NA-NL: *N. limnetica* cultivation on non-autoclaved DPBP medium*.* All results are reported as mean of n ± std, where n = 2 biological replicates × 2 analytical replicates = 4.

The biomass concentration and growth kinetics of each cultivation are shown in Fig. 1 and Table 2 respectively. *N. limnetica* cultivated on autoclaved DPBP medium (A-NL) presented the highest final biomass concentration at 1.363 ± 0.011 g L^−1^ after 8 days of cultivation. The difference between final biomass concentration obtained in autoclaved medium (A-NL) and that obtained in non-autoclaved DPBP cultures (NA-NL, 1.055 ± 0.012 g L^−1^ on day 8) was found to be statistically significant (p < 0.05). On the other hand, saltwater *N. oceanica* barely grew on DPBP medium under axenic conditions (A-NO), obtaining a mere final biomass concentration of 0.028 ± 0.002 g L^−1^ at the end of cultivation period. This was ca. 40 to 50-fold lower than those of *N. limnetica* cultures, indicating DPBP medium in its native state cannot support the growth of marine microalgae *N. oceanica* (Fig. 1). Since DPBP had a much lower salinity compared to seawater (2.47 ± 0.11 parts per thousand vs. 35 parts per thousand), its direct application as growth medium without prior salinity adjustment likely resulted in significant hypotonic stress for the marine microalgal cells, which in turn diminished any potential for cell growth.

**Table 2:** Final biomass concentration, predicted final biomass concentration, and growth rate of *N. oceanica* and *N. limnetica* cultures on autoclaved and non-autoclaved DPBP medium. A-NO: *N. oceanica* cultivation on autoclaved DPBP medium, A-NL: *N. limnetica* cultivation on autoclaved DPBP medium, and NA-NL: *N. limnetica* cultivation on non-autoclaved DPBP medium.

	Final biomass concentration (g L^−1^)	Predicted final biomass concentration Y_m_ (g L^−1^)	Maximum specific growth rate µ_m_ (day^−1^)
A-NO	0.028 ± 0.002	n/a*	n/a
A-NL	1.363 ± 0.011^a^	1.371 ± 0.066	0.256 ± 0.042
NA-NL	1.055 ± 0.012^b^	1.090 ± 0.103	0.148 ± 0.032

*n/*a* Curve cannot be fitted using the selected growth model.

All results are reported as mean of n ± std. n = 2 biological replicates × 2 analytical replicates = 4. Statistical test was applied on the differences in final biomass concentration between A-NL and NA-NL. Since the difference was found to be statistically significant (p < 0.05), it was denoted with different letters in the column (a or b).

### Algae–bacteria population dynamics

Microalgae–bacteria cell population dynamics were determined using flow cytometry coupled with SG fluorescence staining. Microalgal cells in the culture were able to emit both SG fluorescence and autofluorescence after SG staining, while bacterial cells in the culture only emitted SG fluorescence after staining, allowing the sub-populations to be discriminated from each other and thus quantified based on their distinct signal counts. Microalgal cell density was assumed to be equivalent to the autofluorescence signal, while bacterial cell density was calculated as the difference between total SG fluorescence and SG fluorescence associated only with microalgal cells.

Cell density of microalgal cells and bacterial cells in the beginning and at the end of the 8-day cultivation are shown in Table 3. *N. oceanica* culture (A-NO) barely experienced any increase in microalgal cell density (from 1.30 × 10^5^ cells mL^−1^ on day 0 to 2.64 × 10^5^ cells mL^−1^ on day 8), which was in agreement with biomass concentration results as measured by filtration and oven-drying in the previous section, confirming that the species was simply not able to grow on DPBP medium in its native state. For *N. limnetica* cultures, microalgal cell density displayed a significant increase in both non-autoclaved medium and autoclaved medium, rising from ca. 9.5 × 10^4^ cells mL^−1^ on day 0 to 1.10 × 10^7^ cells mL^−1^ (NA-NL) and 1.43 × 10^7^ cells mL^−1^ (A-NL) on day 8.

**Table 3:** Initial cell density and final cell density of microalgal cells and bacterial cells in each culture type. A: autoclaved DPBP control, NA: non-autoclaved DPBP control, A-NO: *N. oceanica* cultivation on autoclaved DPBP medium, A-NL: *N. limnetica* cultivation on autoclaved DPBP medium, and NA-NL: *N. limnetica* cultivation on non-autoclaved DPBP medium. n.d. = not detected (assumed to be 0).

	Day 0 (Initial)					Day 8 (Final)				
	Microalgal cell density (cells mL^−1^)	Bacterial cell density (cells mL^−1^)	Total cell density (cells mL^−1^)	Microalgal cells/total cells (%)	Bacterial cells/total cells (%)	Microalgal cell density (cells mL^−1^)	Bacterial cell density (cells mL^−1^)	Total cell density (cells mL^−1^)	Microalgal cells/total cells (%)	Bacterial cells/total cells (%)
A	n.d	n.d	n.d	–	–	n.d	5.35 × 10^5^	5.35 × 10^5^	0.00	100.00
NA	n.d	8.31 × 10^5^	8.31 × 10^5^	0.00	100.00	n.d	2.45 × 10^7^	2.45 × 10^7^	0.00	100.00
A-NO	1.30 × 10^5^	3.31 × 10^4^	1.63 × 10^5^	79.71	20.29	2.64 × 10^5^	6.33 × 10^5^	8.97 × 10^5^	29.43	70.57
A-NL	9.22 × 10^4 a^	3.80 × 10^4 a^	1.30 × 10^5^	70.81	29.19	1.43 × 10^7 a^	3.54 × 10^6 a^	1.78 × 10^7^	80.16	19.84
NA-NL	9.48 × 10^4 a^	8.46 × 10^5 b^	9.41 × 10^5^	10.08	89.92	1.10 × 10^7 b^	9.72 × 10^6 b^	2.07 × 10^7^	53.09	46.91

Statistical tests were applied on differences in initial microalgal cell density, initial bacterial cell density, final microalgal cell density, and final bacterial cell density between A-NL and NA-NL. If the values for a particular parameter are significantly different between the two cultures (p < 0.05), they are denoted with different letters in the column (a or b). If the values for a particular parameter are not significantly different between the two cultures (p > 0.05), they are denoted with the same letters in the column (a).

Bacterial population was found to increase prolifically in the non-autoclaved control culture (NA), attaining a ca. 55-fold increase in cell density after 8 days of cultivation (from 8.31 × 10^5^ cells mL^−1^ on day 0 to 2.45 × 10^7^ cells mL^−1^ on day 8). The bacterial population in the NA control culture can be assumed to consist exclusively of contaminating bacteria found in the DPBP medium. Expectedly, almost all of the contaminating bacteria in the DPBP medium were killed off during sterilisation process, with the autoclaved control culture (A) registering bacterial density that was below detectable level on day 0.

Bacterial cells in *N. limnetica* culture cultivated on non-autoclaved DPBP medium (NA-NL) can be assumed to comprise both contaminating bacteria in the DPBP medium and native bacteria associated with the microalgal phycosphere. Bacterial cell density in the NA-NL culture grew rapidly from 8.46 × 10^5^ cells mL^−1^ on day 0 to 9.72 × 10^6^ cells mL^−1^ on day 8, albeit reaching a final concentration that was 40% lower than the level of pure bacteria obtained in the non-autoclaved NA control (2.45 × 10^7^ cells mL^−1^)_._ Despite starting at a lower cell density than bacterial cells in day 0, microalgal cells in the NA-NL culture were able to achieve dominance by the end of cultivation, attaining a final cell density of 1.10 × 10^7^ cells mL^−1^ and occupying 53.09% of the total cell density in the culture at the end of the cultivation. This suggests that *N. limnetica* cells were able to establish synergistic relationships with the bacterial cells in the culture, competing for the same pool of available nutrients in a mutually beneficial manner that enabled concurrent growth of both populations.

Since bacterial cells in *N. limnetica* culture cultivated on autoclaved DPBP medium (A-NL) comprised only native/phycospheric bacteria, they started off with a smaller population compared to the culture on non-autoclaved DPBP medium (NA-NL) on day 0 and consequently grew to a lesser extent. The final bacterial cell density of the A-NL culture was 3.54 × 10^6^ cells mL^−1^, 64% less than that obtained at the end of cultivation for NA-NL culture (9.72 × 10^6^ cells mL^−1^). In this case, *N. limnetica* cells were well adapted for symbiotic growth with their own phycospheric bacteria, comfortably retaining dominance in the A-NL culture (final microalgal cell density = 1.43 × 10^7^ cells mL^−1^) and occupying 80.16% of total cell density by the end of cultivation.

These results revealed that in both non-autoclaved (NA-NL) and autoclaved DPBP medium (A-NL), bacterial population grew in parallel with the *N. limnetica* population. In both cases, the microalgae cells were able to gain dominance, suppressing bacterial proliferation as observed in the control culture (NA) and preventing bacterial takeover.

### Bacterial isolation and microscopy

**Figure 2:**
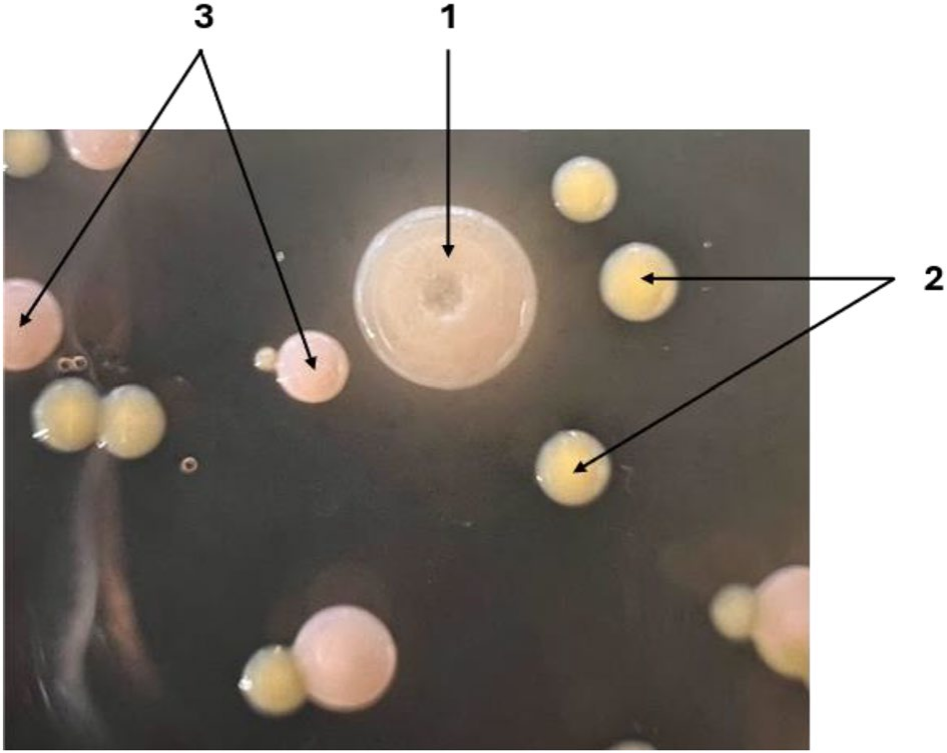
Agar plate of isolated bacteria from *N.limnetica* cultures cultivated on DPBP medium. Three different types of coloured bacterial colonies were observed. (1) ivory-white-coloured colonies comprising gram-negative bacilli, (2) yellow-coloured colonies comprising gram-negative cocci, and (3) orange-coloured colonies comprising gram-positive bacilli.

**Figure 3:**
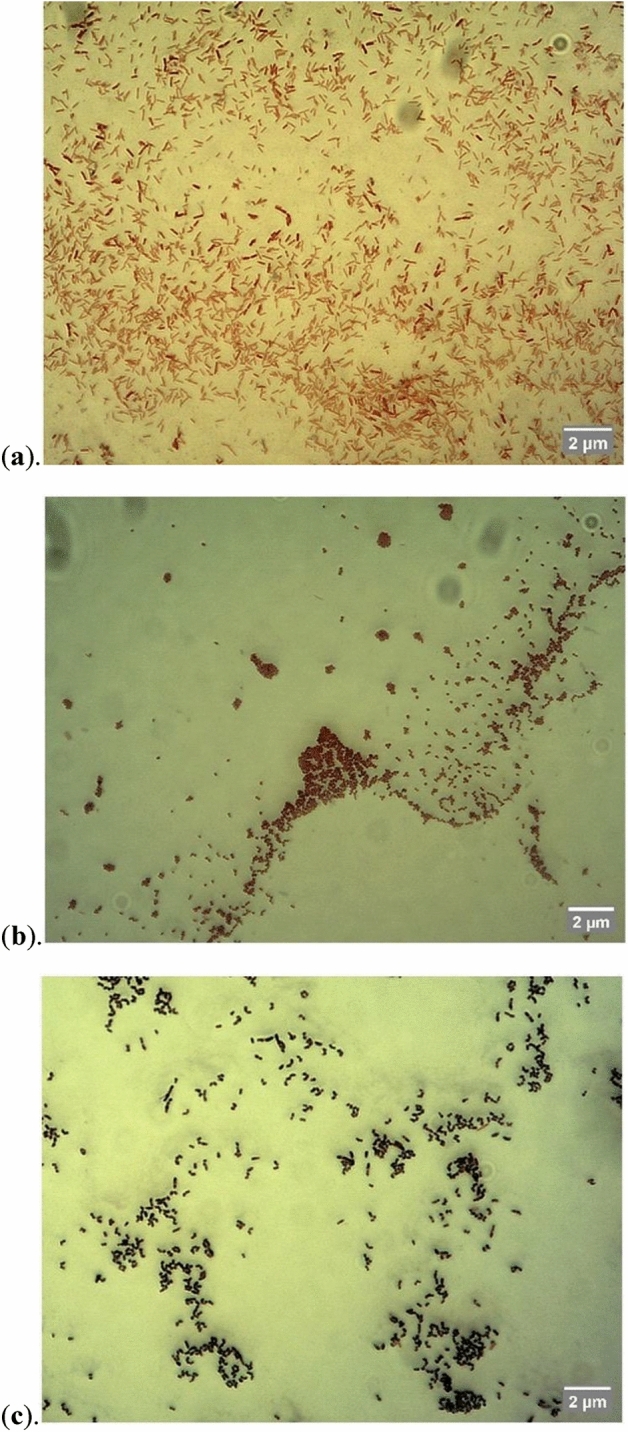
Microscopic images of isolated bacterial cells from *N.limnetica* cultures cultivated on DPBP medium. (**a**) Type 1: gram-negative bacilli, (**b**) type 2: gram-negative cocci, and (**c**) type 3: gram-positive bacilli which joined together to form long-chain rod structures.

Three different bacterial strains were successfully isolated from the NL cultures, each forming a different type of colony on agar plates (Fig. 2). Morphological analysis of the bacterial cells using microscopy + staining showed that bacterial cells in the ivory-white circular and smooth-edge colony (type 1 colony) were gram-negative and rod-shaped with an average length of 0.43 ± 0.03 μm (Fig. 3a). Bacterial cells in the yellow circular smooth-edge colony (type 2 colony) existed in clusters, were gram-negative and cocci-shaped with an average diameter of 0.0022 ± 0.0003 μm (Fig. 3b). The bacterial cells in the orange-pigmented colony (type 3 colony) were organised in chains, gram-positive, and rod-shaped with an average length of 0.41 ± 0.02 μm (Fig. 3c).

The same bacterial strains were isolated from both autoclaved (A-NL) and non-autoclaved DPBP cultures (NA-NL). Such findings were unexpected given the origins of bacterial community in the two cultures, where A-NL culture was anticipated to contain only native bacteria affiliated with the phycosphere, while NA-NL culture was foreseen to contain both contaminating bacteria from the DPBP medium and phycospheric bacteria. In this case, bacterial cells originating from contamination in the medium appeared to be unable to grow on the marine MB agar used for bacterial isolation. All three of the isolated bacterial strains were thus likely native bacteria affiliated with *N. limnetica* phycosphere. To the best of our knowledge, this is the first time that native bacteria associated with *N. limnetica* cells have been isolated, representing a step forward in their identification and possible exploitation for growth enhancement of the species.

### Lactose assimilation and β-galactosidase production

**Figure 4:**
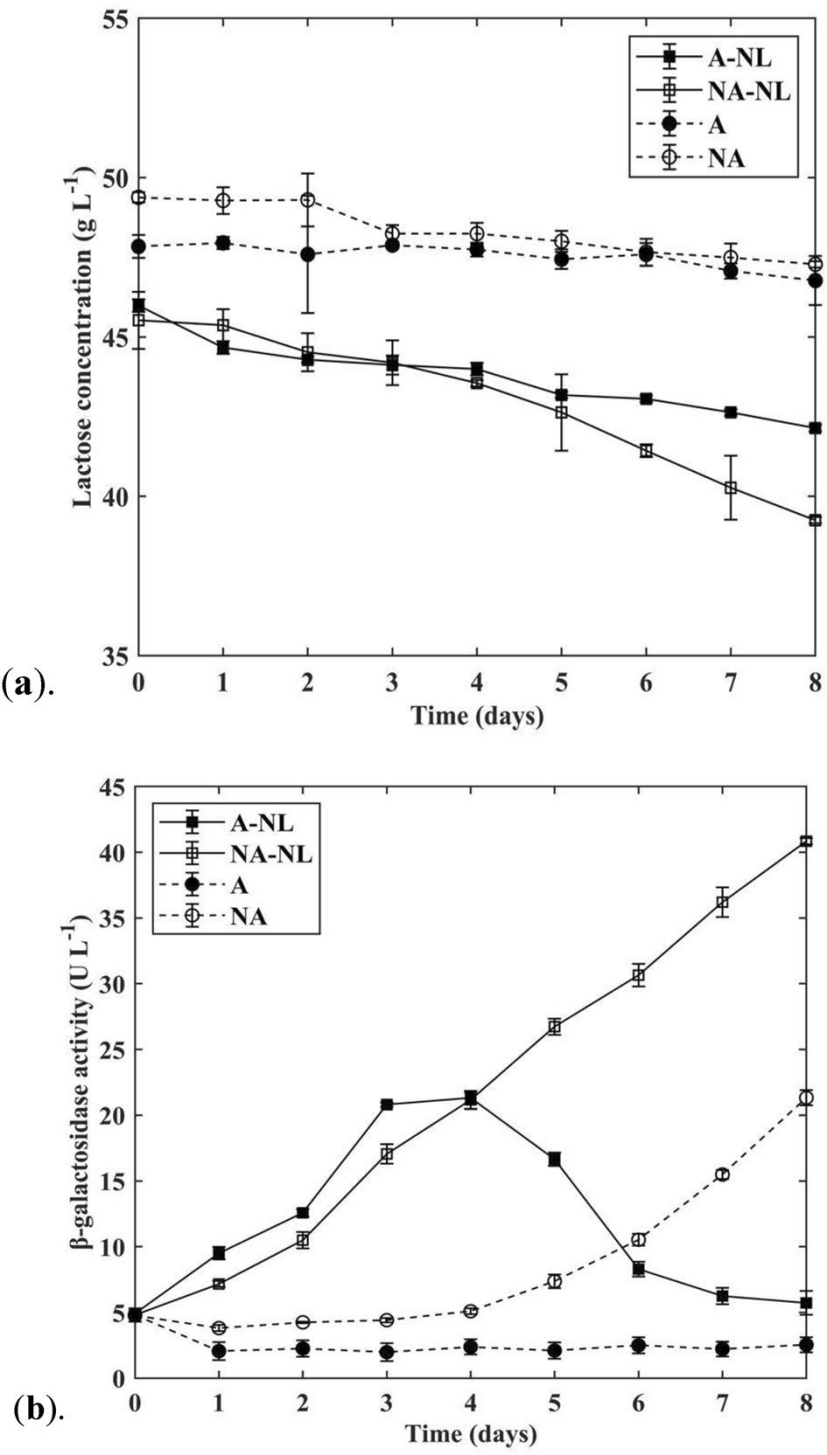
(**a**) Lactose metabolization and (**b**) level of extracellular β-galactosidase secretion in *N. limnetica* and control cultures cultivated on DPBP medium. A: autoclaved DPBP control, NA: non-autoclaved DPBP control, A-NL: *N. limnetica* cultivation on autoclaved DPBP medium, and NA-NL: *N. limnetica* cultivation on non-autoclaved DPBP medium. All results are reported as mean of n ± std, where n = 2 biological replicates × 2 analytical replicates = 4.

Lactose metabolization was observed in both *N. limnetica* cultures (Fig. 4a), with A-NL and NA-NL cultures showing 8.34% and 13.77% reduction in lactose concentration respectively. Glucose and galactose were not detected (below 0.1 g L^−1^) at any time points during the cultivation period. These results exemplified that *N.limnetica* cells were able to assimilate lactose. The absence of any monosaccharide accumulation in the culture medium indicated that the cells were either a) able to assimilate lactose directly into biomass as a disaccharide molecule or b) had the capacity to secrete extracellular β-galactosidase to hydrolyse lactose and then absorb the resultant monosaccharides derived from lactose degradation. In addition, ca. 4% reduction in lactose concentration was also observed in non-autoclaved control samples (NA), revealing that contaminating bacteria from DPBP medium also had the capacity to assimilate lactose, albeit to a much lower extent compared to the microalgal-bacterial consortium in the microalgal culture.

To better understand the pathway of lactose utilisation in *N. limnetica*, β-galactosidase presence in the medium of the cultures was assayed. As shown in Fig. 4b, extracellular β-galactosidase was expressed in the medium, confirming that the cells metabolised lactose through exogenous enzymatic activity followed by absorption of resultant monosaccharides rather than direct absorption of the disaccharide molecules followed by internalised enzyme breakdown into monosaccharides. The cells were then able to rapidly assimilate the monosaccharide molecules derived from the hydrolysis process, thus ensuring that there is no accumulation of any monosaccharides in the medium.

In the A-NL cultures, a marked rise in β-galactosidase activity was observed at the early stage of cultivation. This was then followed by a plateau on day 3–4 at 21.07 ± 0.41 U L^−1^ and a subsequent rapid decline towards the end of cultivation (Fig. 4b). The A-NL culture consisted of the microalgal cells growing in symbiotic cohabitation with native/phycospheric bacterial cells. Even though the source of the β-galactosidase secretion cannot be verified without further molecular investigation (e.g., RT-qPCR), it can be assumed that both microalgal and phycospheric bacterial cells, in this case, worked in partnership to produce β-galactosidase to break down lactose into digestible monosaccharides. The decrease in enzymatic activity towards the end of the growth cycle can be likely attributed to a slowdown in microalgal metabolic rates as nutrients in the medium (e.g., nitrogen) became depleted and the cells prepared to enter stationary phase.

Unlike the A-NL cultures, the NA-NL cultures did not experience enzymatic peak performance. Instead, β-galactosidase activity was found to continue to increase throughout the entire cultivation period, resulting in the highest enzymatic activity observed across all cultures at 40.84 ± 0.23 U L^−1^ on day 8 of cultivation (Fig. 4b). The trend of enzymatic activity in the NA-NL culture appeared to correlate well with that of biomass concentration in Fig. 1, with values for both parameters experiencing continuous increase throughout cultivation. This suggests constant replenishment of enzymes by newly formed biomass in the culture.

Interestingly, a continuous increase in enzymatic activity during the cultivation period was also found in the non-axenic control sample (NA), mirroring the pattern attained in the NA-NL cultures. This validated our earlier results that contaminating bacteria in the DPBP medium also had the capacity to generate extracellular β-galactosidase and assimilate lactose as a carbon source. The findings also provided an explanation for the divergence in the profile of enzymatic activity between the NA-NL and A-NL cultures. Unlike the A-NL cultures which contained only symbiotic bacteria, the NA-NL cultures had a more diverse set of bacterial population, which included contaminating bacteria from DPBP medium. These foreign bacteria led to the replenishment of enzymes in the NA-NL cultures and thus contributed to the continuous increase in enzymatic activity throughout cultivation. However, the highest enzymatic activity in non-axenic control samples (NA) was two-fold lower than that in NA-NL cultures, despite having the highest final bacterial cell density across all cultures at 2.5-fold greater than that in NA-NL cultures (Table 3). The results indicated that contaminating bacterial cells alone from the DPBP medium were inefficient in producing β-galactosidase. In the NA-NL cultures, microalgal cells, phycospheric bacterial cells and contaminating bacterial cells all worked in tandem to produce β-galactosidase, resulting in the highest formation of extracellular enzymes.

### Bioremediation of nitrogen and phosphate

**Figure 5:**
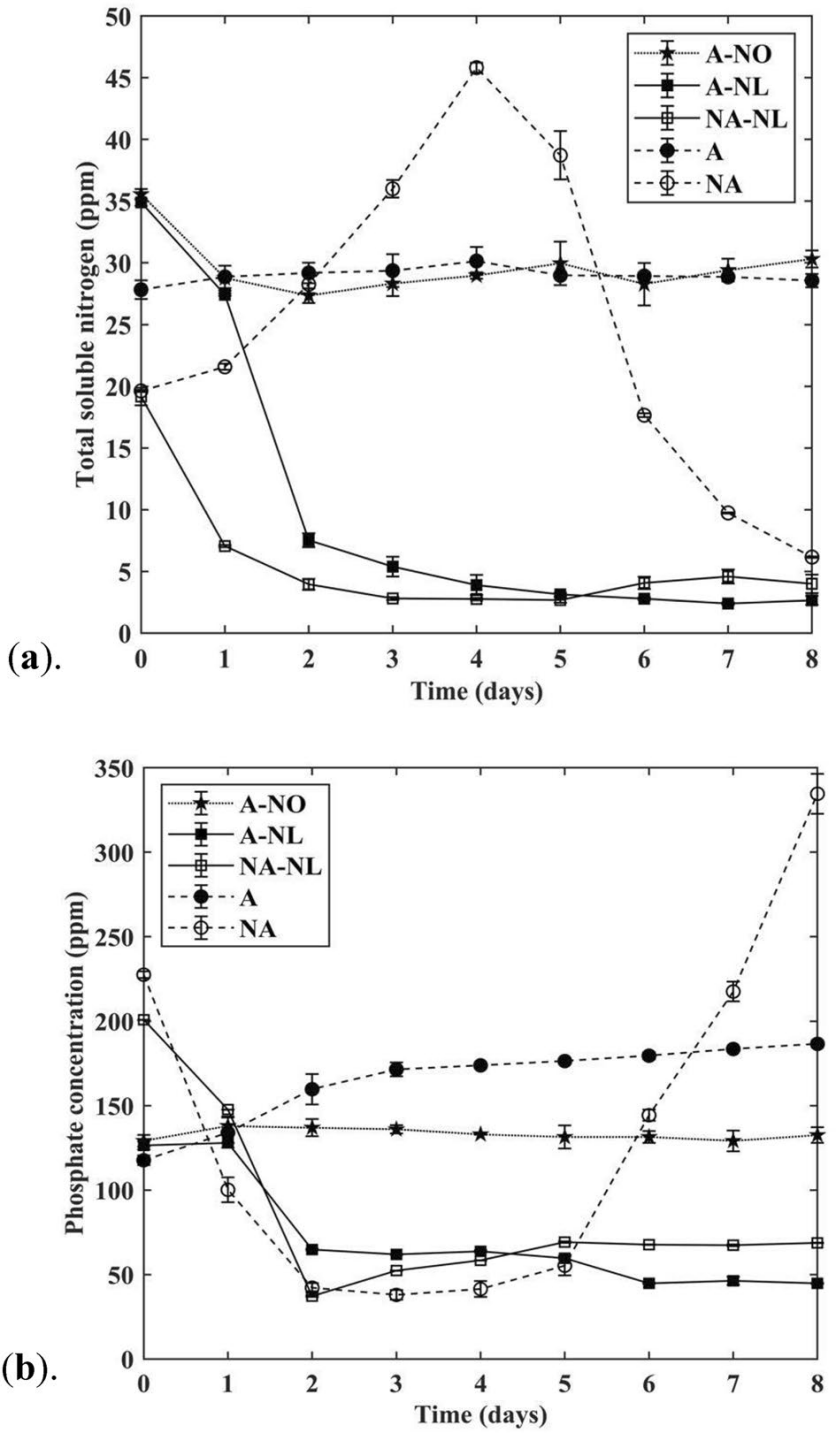
(**a**) Total soluble nitrogen concentration and (**b**) phosphate concentration of *N. limnetica* cultures and control cultures. A: autoclaved DPBP control, NA: non-autoclaved DPBP control, A-NO: *N. oceanica* cultivation on autoclaved DPBP medium, A-NL: *N. limnetica* cultivation on autoclaved DPBP medium, and NA-NL: *N. limnetica* cultivation on non-autoclaved DPBP medium. All results are reported as mean of n ± std, where n = 2 biological replicates × 2 analytical replicates = 4.

Total soluble nitrogen and phosphate removals were used to assess the capacity of *N. limnetica* cultures for the bioremediation of DPBP medium. As shown in Fig. 5a, total soluble nitrogen was depleted by ca. 80% in both *N. limnetica* cultures (A-NL and NA-NL) within two days. A similar pattern was observed for phosphate bioremediation (Fig. 5b), with both NA-NL and A-NL cultures reaching > 80% depletion of phosphate within two days.

A higher final biomass concentration was obtained in the A-NL cultures (Fig. 1, Table 2), despite the more promising lactose utilisation (Fig. 4) observed in the cultures cultivated on non-autoclaved DPBP medium (NA-NL). This indicates that contaminating bacteria in the DPBP medium likely competed with microalgal cells for nutrients and ended up ‘stealing’ these nutrients to support own growth. *N. limnetica* cells in this study have demonstrated significant resilience against bacterial presence and successfully achieved dominance in non-autoclaved medium possessing a high initial bacterial load (NA-NL culture). There is, however, likely to be a limit to this degree of adaptability. Excessive presence of bacterial cells in the growth medium can be counterproductive to the formation of a mutually beneficial microalgal/bacterial synergy.

As expected, the control culture in the autoclaved medium (A) did not display any detectable microbial growth and hence experienced no reduction in the level of soluble nitrogen and phosphates (Fig. 5a, b). On the other hand, the control culture in the non-autoclaved medium (NA) exhibited an unexpected increase in the level of total soluble nitrogen and phosphate (Fig. 5a, b). This can likely be ascribed to several reasons, including the propagation of extra polymeric substrates (EPS) by contaminating bacterial cells in the DPBP medium and the release of phosphate and nitrogen into the medium facilitated by decomposition of dead bacterial cells.

## Discussion

This study investigated the use of *N. limnetica* for the valorisation of whey powder (or DPBP) into valuable biomass and β-galactosidase enzyme, deriving critical insights in (1) the species capacity to grow on dairy side streams, (2) the need for pre-treatment to manage bacterial contamination, (3) the production level of β-galactosidase and extent of nutrient bioremediation and (4) the roles of microalgae–bacteria interaction in culture performance.

### Capacity to grow on DPBP medium

Freshwater microalgae strain *N. limnetica* was able to grow on DPBP medium without any salinity or nutrient adjustment, reaching a final biomass concentration between 1.06 and 1.36 g L^−1^ within 8 days (Fig. 1), which was ca. twofold higher than those reported in our previous study ^3^ using *N. oceanica* cultivated on lactose-rich standard growth medium.

### Managing bacterial contamination

Cultivation of *N. limnetica* on autoclaved DPBP medium (A-NL) achieved a higher final biomass concentration (by ca. 30%) compared to that on non-autoclaved DPBP medium (NA-NL). This can likely be ascribed to the dual effects incurred by autoclaving on both the nutrient profiles and the initial bacteria cell density (day 0) of the medium. Autoclaving led to a twofold increase in the concentration of total soluble nitrogen and a threefold increase in N:P ratio of the DPBP medium. Nitrogen concentration in a medium plays an important role in microalgae growth and biomass composition^38^, with higher initial nitrogen concentration generally shown to result in rapid cell proliferation and increased biomass concentration^39^. Increased initial bacterial cell density in the non-autoclaved medium led to increased competition for nutrients (i.e., lactose, N and P) which contributed to a lower microalgal biomass production in the non-autoclaved DPBP medium (bacterial cell density on day 0 = 8.46 × 10^5^ cells mL^−1^ for NA-NL culture and 3.80 × 10^4^ cells mL^−1^ for A-NL cultures). In the autoclaved cultures, microalgae cells only had to compete with native/phycosphere bacteria, while cells cultivated on non-autoclaved medium had to overcome competition with both contaminating bacteria in the DPBP medium and native/phycosphere bacteria.

In both A-NL and NA-NL cultures *N. limnetica* cells were able to develop a synergy with the bacterial cells, growing in tandem with the bacterial population and emerging as the dominant population in the cultures by the end of cultivation. The extent of microalgal cell dominance, however, was less pronounced in the non-autoclaved medium compared to the autoclaved medium (Table 3). These results indicate the need for a pre-treatment method to remove contaminating bacteria in the medium prior to microalgal inoculation especially if the system were to be scaled-up and operated continuously/semi-continuously over multiple growth cycles. In this study, autoclaving has been employed as a pre-treatment method. However, non-thermal and less-degradative mechanical methods with lower infrastructure and operating costs (e.g., crossflow microfiltration or multi-step filtration) can be considered for industrial-scale applications.

### Production of β-galactosidase and bioremediation capacity

*N. limnetica* were able to produce extracellular β-galactosidase enzyme when cultivated on dairy medium. In the study, this resulted in a ca. 6 g L^−1^ reduction in lactose concentration in the DPBP medium by the end of cultivation (Fig. 4). No monosaccharides were detected in the medium at any of the time points, suggesting complete uptake of simple sugars derived from lactose breakdown by the cells. This is well aligned with previous results reported for *Nannochloropsis* by Zanette et al.^15^ and Li et al.^3^. The maximum extracellular β-galactosidase activity (40.84 ± 0.23 U L^−1^) measured in this study was similar to those secreted by other microalgae species^3,15,40^, but lower than those previously reported in other studies with bacteria and yeast^41,42^. Studies conducted by Suwal et al.^18^ and Li et al.^3^ reported similar patterns when cultivating *Nannochloropsis* on lactose-enriched standard medium, observing a peak of extracellular β-galactosidase activity roughly midway through cultivation before a sharp decrease. The secretion of microbial-derived enzymes is affected by variations in species and cultivation conditions (e.g., pH, temperature)^43^. Studies conducted by Jurado et al.^44^ and Otieno^45^ indicated that high mineral ion concentration, such as that encountered in dairy waste, could lead to an inhibitory effect on the β-galactosidase secretion by microorganisms.

The origin of β-galactosidase enzyme production in the study was not elucidated. *N. limnetica* cultivated on non-autoclaved DPBP medium (NA-NL) generated the highest amount of exogenous β-galactosidase, followed by *N. limnetica* cultivated on autoclaved DPBP medium (A-NL) and control cultures with no microalgae cells (NA). It can thus be deduced that *N. limnetica* cells and bacterial cells were individually able to produce their own β-galactosidase. Beneficial interaction taking place between microalgal cells and cells of contaminating bacteria synergistically enhanced β-galactosidase in the NA-NL cultures.

### Prospect of *N. limnetica* for dairy side-stream treatment

In all cases, *N. limnetica* exhibited high nutrient removal efficiency, being able to eliminate > 80% of available nitrogen and phosphates in the DPBP medium by the end of cultivation. Overall, the high nutrient removal efficiency and competitive biomass and β-galactosidase productivity found in this study demonstrate the promising nature of freshwater microalgae *N. limnetica* for the valorisation of dairy processing side streams.

### Role of microalgae–bacteria interaction

**Figure 6:**
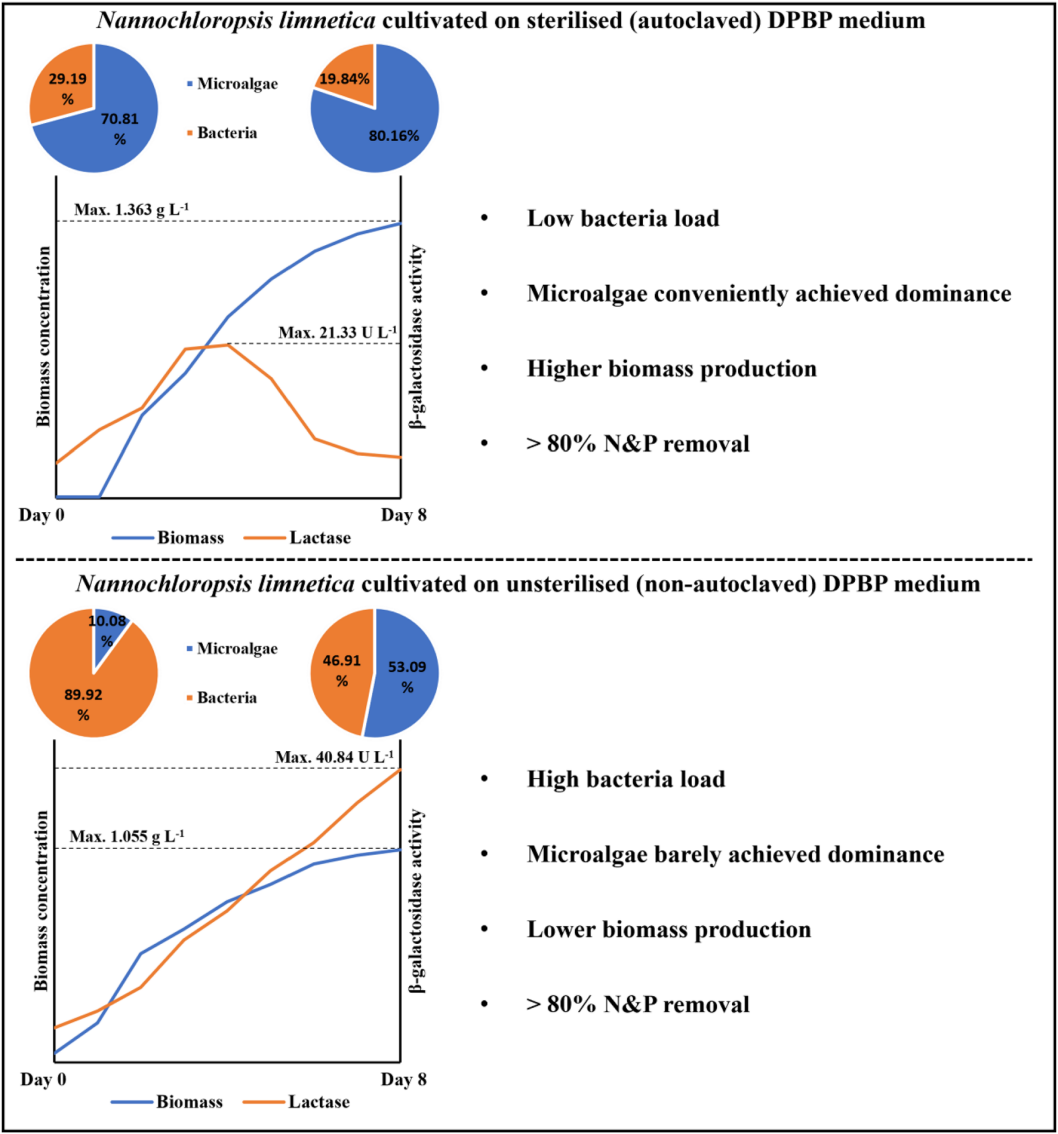
The role of microalgae–bacteria interaction in *N. limnetica* cultures cultivated on DPBP medium in terms of biomass production, enzyme secretion and bioremediation performance.

Figure 6 summarises the effect of microalgae–bacteria interaction in *N. limnetica* culture on biomass production, enzyme secretion, and bioremediation performance. Cultures grown on non-autoclaved DPBP medium had a higher initial bacterial load compared to those grown autoclaved DPBP medium due to the presence of contaminating bacteria from the medium. The increased bacterial presence was found to lead to mixed benefits in microalgal culture performance, increasing the production of β-galactosidase enzymes on one hand, while competing for nutrients with microalgal cells and ultimately leading to a lower final microalgal biomass concentration. In the future, 16 s RNA sequencing and metabolic fingerprinting can be used to identify the bacteria strains isolated from the *Nannochloropsis* culture to obtain a better understanding of the algal–bacterial interactions and harness potential synergy to improve microalgal growth.

Co-occurring bacteria in algal culture had the potential to either benefit or harm algae growth. A study conducted by Lian et al.^46^ indicated that *Alphaproteobacteria* and *Flavobacteriia* were two major classes of phycospheric bacteria isolated from *Nannochloropsis* cultures; two of these strains, *Maritalea porphyrae* and *Labrenzia aggregata*, had been proven to promote *Nannochloropsis* growth, while flavobacterial strains were found to significantly inhibit the microalgae growth. In addition, associated bacterial species belonging to *Proteobacteria* phylum has also been reported in *Nannochloropsis* sp. cultures^47^. These bacteria had denitrifying capabilities^48^ and were able to efficiently metabolise carbon and nitrogen from the culture due to their transporter genes^43,49,50^.

In terms of the contaminating bacteria in DPBP medium, lactobacilli are widely used as a probiotic in yogurt manufacture processing and are thus likely to be one of bacterial phylum present in dairy side streams^51^. According to Maske et al.^43^, lactobacilli also had the capability to produce a range of digestive enzymes, including lactase, proteases and amylases^46,52–54^.

## Conclusion

This study investigated the application of microalgae *Nannochloropsis limnetica* as a green strategy to bioremediate dairy processing by-products (DPBP) and simultaneously co-produce biomass and the valuable enzyme β-galactosidase. *N. limnetica* was able to grow on the whey powder solution without the need for any salt or nutrient addition, reaching a final biomass concentration ranging from 1.06 to 1.36 g L^−1^. The species were able to produce extracellular β-galactosidase in DPBP as part of their lactose metabolization pathways, attaining a maximum enzymatic activity of 40.84 ± 0.23 U L^−1^. The species displayed the capacity to efficiently remove nutrients from DPBP, achieving 80% depletion of both total soluble nitrogen and phosphate from the medium within 2 days.

*Nannochloropsis* cells were able to maintain dominance over bacterial cells throughout all cultivations (between 53 and 80% of total cells). The extent of microalgal dominance over bacterial cells was less prominent in medium that had not been subjected to any pre-treatment compared to medium that had previously been autoclaved. Cultures grown on untreated medium has a high initial bacterial load attributed to the presence of both contaminating bacteria and phycospheric bacteria. High initial bacterial density in the culture was found to have mixed effects on *Nannochloropsis* performance, promoting β-galactosidase synthesis on the one hand while competing for nutrients and retarding biomass production on the other.

In the current study, autoclaving was used to eliminate contaminating bacteria from DPBP medium. The pre-treatment step, however, resulted in inadvertent changes in the N:P profile of the medium. Therefore, other pre-treatment technologies (e.g., crossflow microfiltration) able to remove contaminating bacteria without amending nutrient compositions should be considered in future studies. In addition, future studies should also carry out bacteria identification at either a molecular level or using metabolic fingerprinting to gain a better understanding of the synergy between microalgal and bacterial cells. Overall, this study demonstrated the promising nature of freshwater *Nannochloropsis* in recovering nutrients and producing valuable enzyme from dairy side streams.

## Data Availability

The datasets analysed during the present study are available from the corresponding author on reasonable request.
